# Identification of differentially-expressed genes by DNA methylation in cervical cancer

**DOI:** 10.3892/ol.2015.2917

**Published:** 2015-01-29

**Authors:** HEUN-SIK LEE, JUN HO YUN, JUNGHEE JUNG, YOUNG YANG, BONG-JO KIM, SUNG-JONG LEE, JOO HEE YOON, YONG MOON, JEONG-MIN KIM, YONG-IL KWON

**Affiliations:** 1 Center for Genome Science, Korea National Institute of Health, Osong Health Technology Administration Complex, Cheongju, Chungcheongbuk-do 363-951, Republic of Korea; 2 Macrogen Inc., Seoul 153-023, Republic of Korea; 3 Center for Women’s Disease, Department of Biological Science, Sookmyung Women’s University, Seoul 140-742, Republic of Korea; 4 Department of Obstetrics and Gynecology, Saint Vincent’s Hospital, the Catholic University, Suwon, Gyeonggi-do 442-723, Republic of Korea; 5 Department of Public Health Administration, Namseoul University, Cheonan, Chungcheongnam-do 331-707, Republic of Korea; 6 Department of Obstetrics and Gynecology, Kangdong Sacred Heart Hospital, Hallym University Medical Center, Seoul 134-701, Republic of Korea

**Keywords:** DNA methylation, RNA expression, integrated analysis, epigenetics, cervical cancer

## Abstract

To identify novel cervical cancer-related genes that are regulated by DNA methylation, integrated analyses of genome-wide DNA methylation and RNA expression profiles were performed using the normal and tumor regions of tissues from four patients; two with cervical cancer and two with pre-invasive cancer. The present study identified 19 novel cervical cancer-related genes showing differential RNA expression by DNA methylation. A number of the identified genes were novel cervical cancer-related genes and their differential expression was confirmed in a publicly available database. Among the candidate genes, the epigenetic regulation and expression of three genes, *CAMK2N1*, *ALDH1A3* and *PPP1R3C*, was validated in HeLa cells treated with a demethylating reagent using methylation-specific polymerase chain reaction (PCR) and quantitative PCR, respectively. From these results, the expression of the *CAMK2N1*, *ALDH1A3* and *PPP1R3C* genes are were shown to be suppressed in cervical cancers by DNA methylation. These genes may be involved in the progression or initiation of cervical cancer.

## Introduction

Cervical cancer is an major cause of mortality for females, with the worldwide incidence of cervical cancer reaching 454,000 cases per year in 2010 (1). Although persistent infection by human papillomavirus (HPV) is necessary to cause cervical cancer, it is not sufficient for disease progression (2). Other etiological factors, including smoking, are also known to contribute to cervical cancer (3). Genetically, the HPV ‘early’ proteins, E6 and E7, are the primary oncoproteins involved in cancer progression and interact with several cellular proteins, including tumor protein 53 (TP53), retinoblastoma 1 (RB1), Bcl-2 antagonist killer (BAK), Fas-associated death domain (FADD) and insulin-like growth factor-binding protein 3 (IGFBP3), involving cell adhesion, apoptosis and the cell cycle (4–6).

DNA methylation is a well-known epigenetic marker, as is histone modification (7). The DNA hypomethylation and hypermethylation of several cancer-related genes have been extensively studied (8–10). In cervical cancer, DNA methylation also affects the expression of several genes, including *CDH1*, *DAPK*, *TIMP-3*, *p16 (INK4a)*, *FHIT* and *RASSF1A* (11,12). Unlike studies involving targeted candidate gene approaches, there have been few reports regarding genome-wide methylation profiles for investigating the methylation status of the whole genome region by using a high-density microarray-based platform. Recently, two differentially-methylated regions, *COL25A1* and *KATNAL2*, were reported as candidate biomarkers in a genome-wide survey using a high-density microarray system (13). Furthermore, a few studies have conducted integrated analyses of DNA methylation and RNA expression in breast and ovarian cancer, but not in cervical cancer (14,15).

In the present study, genome-wide DNA methylation was examined, RNA expression profiles were analyzed and an integrated analysis of these two profiles was conducted to identify differentially-expressed DNA methylation-regulated genes that are involved in cervical cancer. Finally, the study identified several novel genes associated with cervical cancer that showed differential RNA expression by DNA methylation in tumor regions and validated the epigenetic regulation of selected genes by treatment with a demethylating reagent of the cervical cancer cell line HeLa.

## Materials and methods

### Tissues, cell lines and reagents

Normal and tumor regions of cervical tissues were biopsied from four patients, two pre-invasive cancer subjects (CIN III and CIS), and two patients with cervical cancer (CxCa Ib and CxCa IIa), and stored at the Kangdong Sacred Heart Hospital (Hallym University, Seoul, South Korea) following international ethical guidelines. All the patients provided written informed consent. This study was approved by the Kangdong Sacred Heart Hospital Institutional Review Board. Genomic DNA and RNA were purified from frozen tissues. HeLa cells were obtained from the American Type Culture Collection (Manassas, VA, USA) and cultured in Dulbecco’s modified Eagle’s medium (Gibco-BRL, Grand Island, NY, USA) supplemented with 10% fetal bovine serum, 100 U/ml penicillin and 100 μg/ml streptomycin at 37°C in a humidified CO_2_ incubator.

### Global gene expression profiles

Total RNA was extracted using TRIzol^®^ (Invitrogen Life Technologies, Carlsbad, CA, USA) and further purified using RNeasy columns (Qiagen, Hilden, Germany) according to the manufacturer’s instructions. RNA purity and integrity were evaluated by gel electrophoresis and the ratio of absorbance at 260 and 280 nm using the NanoDrop^®^ ND-1000 (Thermo Scientific, Waltham, MA, USA). Samples were further analyzed on an Agilent 2100 Bioanalyzer (Agilent Technologies, Santa Clara, CA, USA). Microarray analysis using human HT-12 expression v.4 bead array (47,000 probes; Illumina, Inc., San Diego, CA, USA) was carried out by Macrogen Co. (Seoul, South Korea) following the manufacturer’s instructions. Raw data were extracted using the software provided by the manufacturer [Illumina GenomeStudio (v2011.1) Gene Expression Module (v1.9.0)] and filtered if the P-value was <0.05, similar to signal to noise ratio, in ≥50% of the samples analyzed. Signal values of the selected genes were transformed using logarithms and normalized based on the quantile method. The statistical significance of the expression data was determined using an independent Student’s t-test and fold change, in which the null hypothesis was such that no difference existed between the two groups. The false discovery rate was controlled by adjusting the P-value using the Benjamini-Hochberg algorithm. Gene enrichment and functional annotation analysis was performed using Panther DB (http://www.pantherdb.org/). All data analysis and visualization of differentially expressed genes was conducted using The R project (www.r-project.org).

### Genome-wide DNA methylation profiles

Genomic DNA was purified using the DNeasy mini-kit (Qiagen), and sample quality was evaluated using the NanoDrop ND-1000. Intact genomic DNA was diluted to 50 ng/μl based on Quant-iT Picogreen (Invitrogen Life Technologies) quantitation. Microarray analysis using Infinium Human Methylation 450K BeadChip (Illumina, Inc.) was carried out by Macrogen Co., followed by the manufacturer’s instructions. Image processing and raw data extraction were performed using GenomeStudio software [Illumina GenomeStudio (v2011.1) Methylation Module (v1.9.0)] following the manufacturer’s instructions. The β-value was calculated by subtracting the background signals using negative controls on the array and determining the ratio of the methylated signal intensity against the sum of the methylated and unmethylated signals. A β-value of 0–1.0 was reported as a significant percentage of methylation, 0–100%, respectively, for each CpG site (16). Array CpG probes with a detection P-value of ≥0.05, similar to the signal to noise ratio, in >50% samples were filtered out. A filtering criterion was applied for data analysis; a good signal value was required to obtain a detection P-value of <0.05. Filtered data was normalized using the quantile method. Differential methylation was determined based on |Δ_mean| ≥0.2 (difference in methylation signal = average β of case - average β of control).

### Integrated analysis of DNA methylation and gene expression and selection of differentially-expressed mRNA and CpGs

Statistically significant mRNA and CpGs were identified by |fold change| ≥2 and |Δ_mean| ≥0.2, respectively. Next, putative mRNA-CpG target pairs with regulatory associations were extracted. This approach assumed that DNA methylation is negatively correlated with the mRNA expression of its targets. Negative correlation was identified between putative pairs of mRNA and CpGs. The novelty of selected genes with cervical cancer was confirmed in cervical cancer gene database (CCDB; http://crdd.osdd.net/raghava/ccdb) (17). To reduce the bias of the present data, which came from a limited number of samples, the expression of selected genes was confirmed in a publicly accessible microarray database, the gene expression database across normal and tumor tissues (GENT; http://medicalgenome.kribb.re.kr/GENT) (18). All statistical data analysis was conducted using R 2.15.1 (www.rproject.org).

### Functional annotation clustering of selected genes and clustering and functional annotation of Database of Annotation, Visualization and Integrated Discovery (DAVID) analysis

Hierarchical clustering of DNA methylation and RNA expression profiles was conducted using Cluster 3.0 (19). Input data was analyzed by array mean centering and followed by average linkage clustering using correlation (centered) similarity metrics. Clustering results were viewed using TreeView version 1.60 (Eisen Lab, Berkeley, CA, USA). Functional annotation clustering and pathway analysis were conducted using DAVID (20). Functional clustering analysis of all selected genes was performed using medium classification stringency (κ similarity: Similarity term overlap, 3; threshold, 0.5; Classification: Initial and final group membership, 3 and multiple linkage threshold, 0.5).

### Demethylation by treatment with 5-aza-2′-deoxycytidine

HeLa cells (4.0×10^4^) were plated in a six-well plate and treated with various concentrations (0, 5, 10 and 20 μM) of 5-aza-2′-deoxycytidine as decitabine (Selleckchem; Houston, TX, USA) for 96 h. Total DNA and RNA were isolated using the DNeasy and RNeasy mini kits, respectively.

### Reverse transcription (RT) quantitative polymerase chain reaction (PCR) analyses

RT was performed with 2 μg total RNA using the First-Strand cDNA Synthesis kit (Invitrogen Life Technologies) following previously described procedures (21). To quantify the expression levels of the selected genes, quantitative PCR analysis was performed using the first cDNAs as templates and a specific primer set for each gene, *ALDH1A3* (P290199), *PPP1R3C* (P131676) and *CAMK2N1* (P229340) (Bioneer Co., Daejeon, Korea) following the manufacturer’s instructions. Gene amplification was performed using the ABI^®^ PRISM 7900HT Sequence Detection System (Applied Biosystems Life Technologies, Foster City, CA, USA) in a 20 μl reaction mixture containing 2 μl cDNA template (80 ng), 2.5 μl of each primer, 3 μl distilled water and 10 μl 2X Power SYBR Green PCR Master mix (Applied Biosystems Life Technologies), which included a dNTP mixture. PCR conditions were as follows: Initial denaturation at 95°C for 10 min, followed by 40 cycles of 95°C for 10 sec and 60°C for 30 sec. Sequence Detection System (SDS) Software version 2.4 (Applied Biosystems Life Technologies) was used to calculate Ct values for all genes. Relative expression was calculated using the 2^−ΔΔCt^ method.

### Methylation-specific (MS)-PCR analysis

Genomic DNA was modified using the EpiTect Bisulfite kit (Qiagen), which converts cytosine residues to uracil, but does not affect 5-methylcytosine, thus allowing identification of methylated sequences in the DNA by subsequent MS-PCR analysis. For MS-PCR analysis, MS primers (MSP) and unmethylation-specific primers were designed using methylation identification software (22). PCR was then performed in a final volume of 50 μl using 100 μg bisulfite modified DNA and 10 pmol of each primer using the EpiTect MSP kit (Qiagen) under the following conditions: Initial denaturation at 95°C for 5 min, 35 cycles of 95°C for 30 sec, 55°C for 30 sec and 72°C for 30 sec, and a final extension step of 72°C for 5 min. PCR products (20 μl) were visualized on 1.8% agarose gels using the Gel Documentation System (AE-9000 E-graph; ATTO Corporation, Tokyo, Japan).

## Results

### Identification of novel genes regulated by DNA methylation in cervical cancer

Data from DNA methylation and RNA expression was analyzed using an integrated approach and strong cut-off values to overcome the limited number of samples. For differentially-methylated genes in cervical cancer tissues, methylation loci with an absolute value ≥0.2 of the Δ_mean in the methylation profile were selected from the data set. To select genes showing differential expression in cancer tissues, genes with ≥2-fold changes in cancer tissues were selected. The data from selected methylated loci and RNA expression were then merged. To prevent the selection of genes with individual variations, genes showing no methylation in the DNA methylation profiles and no change in the expression profiles in one of two individuals were removed. Finally, nine genes that were downregulated by hypermethylation and four genes that were upregulated by hypomethylation in the cancer tissues were selected. Five genes that were downregulated by hypermethylation and one gene that was upregulated by hypomethylation in the pre-cancer tissues were also selected (Table I).

### In silico analysis of the association of selected genes with cervical cancer

Next, the selected genes were analyzed using a publicly available database. Firstly, it was determined whether the genes had been reported as cervical cancer-related genes in the CCDB. As shown in Table I, only one of the downregulated genes in the tumor tissues, *PPP1R3C*, had previously been reported to be downregulated in cervical cancer. Almost all the selected genes were identified as novel genes associated with cervical cancer. Secondly, to elucidate whether the selected genes are expressed differentially in cervical cancer, the RNA expression patterns of genes in the tissue samples from patients with cervical cancer were analyzed using GENT. Four genes, *PPP1R3C*, *PHF21B*, *TPM1* and *PPP1R14A*, were highly downregulated in the tumor tissues compared with the normal tissues. In addition, three genes, *MAP1LC3A*, *PRRX1* and *SYT11*, were moderately downregulated in the tumor tissues. Other genes, *CAMK2N1* and *ALDH1A3*, showed no change or only minimal upregulation in the tumor tissues. These expression patterns were similar to the RNA expression data. The same analysis was conducted using genes upregulated by hypomethylation in cancer and using genes up- or downregulated in pre-cancer. Seven genes were determined as novel genes associated with cervical cancer, since they had not been reported in the CCDB. The majority of those genes, excluding *FAT1*, which appeared as not applicable in GENT, were up- or downregulated in the tumor tissue samples of patients with cervical cancer. GENT and CCDB data supported the observed association of the selected genes with cervical cancer.

### Functional annotation clustering of selected genes and clustering and functional annotation of DAVID analysis

**Figure 1:**
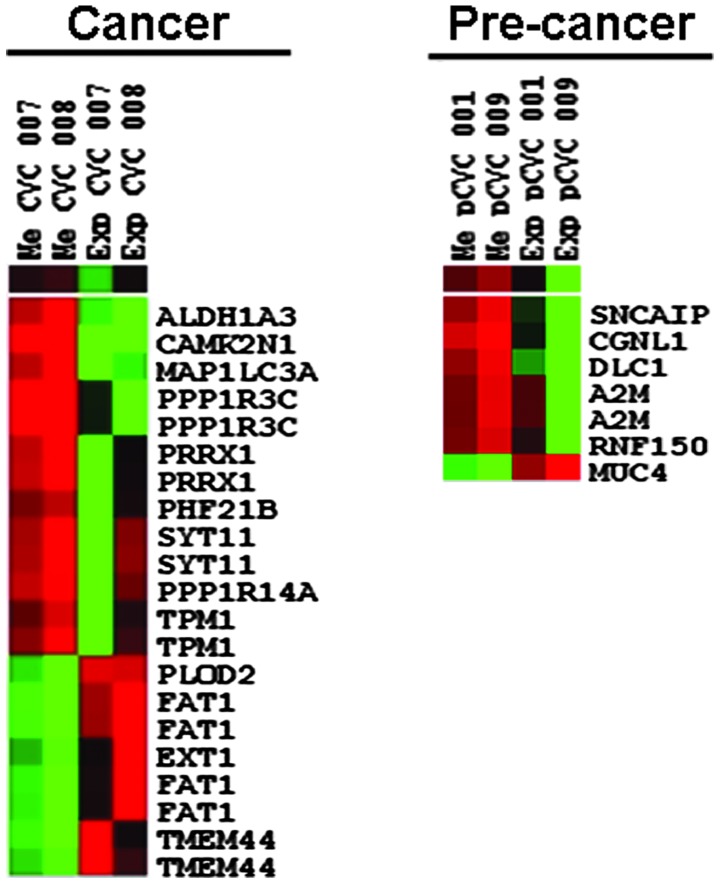
Heatmaps of differentially-expressed genes and methylated loci. There are 19 significantly up- or downregulated genes and 28 methylated loci revealing differential mRNA expression and methylation profiles in the cervical cancer tissues. Different numbers for expression and methylation profiles are observed since some of the genes have two or more methylation loci. Red and green colors indicate the extent of the profiles in the cancer tissues versus that of the normal tissues of each patient sample. For example, red color in methylation profiles and green color in expression profiles show the hypermethylation and downregulation in cancer versus normal tissues, respectively.

To confirm the negative correlation between DNA methylation and RNA expression profiles associated with the progression of cervical cancer, 19 genes that were selected on the basis of integrated analysis were clustered. Common methylation and expression patterns were visualized by unsupervised hierarchical clustering using an average linkage clustering method. As shown in Fig. 1, a separate cluster was formed by methylation and expression data. A negative correlation of methylation and expression was observed using TreeView. Functional annotation clustering was conducted using DAVID with default conditions to analyze the biological significance of the 19 selected genes. This showed that the selected genes were classified into five clusters to be enriched in the cell junction, membrane/plasma membrane, cytoskeletal part, glycoprotein and ion/metal binding (Table II).

### RT-PCR analysis for the validation of DNA methylation by MSP and RNA expression using the cervical cancer cell line HeLa, following treatment with 5-aza-2′deoxycytidine

**Figure 2:**
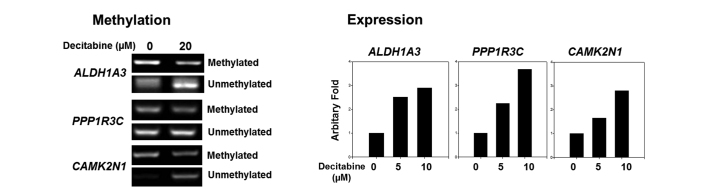
Validation of epigenetic regulation of selected genes in cervical cancer cells. HeLa cells were plated and treated with various concentrations of 5-aza-2′-deoxycytidine as decitabine for 96 h. Purified genomic DNA was modified by converting cytosine residues to uracil, and methylation-specific-PCR analysis was conducted with methylation- and unmethylation-specific primers for each gene. Reverse transcription was performed with total RNA, and quantitative PCR analysis was performed. PCR, polymerase chain reaction.

To further confirm whether the selected genes were regulated by DNA methylation, the HeLa cells were treated with a demethylating reagent, and the methylation status and RNA expression level was measured by MS-PCR using each primer set (Table III) and RT-quantitative PCR analysis, respectively. Methylation of selected genes, *CAMK2N1*, *ALDH1A3* and *PPP1R3C*, was found to be decreased, while demethylation was increased following treatment with the demethylating reagent, as expected. Additionally, RNA expression was increased dose-dependently as a result of the decreased methylation status (Fig. 2). These results indicated that the genes were originally methylated in cervical cancer cells and showed increased demethylation followed by increased RNA expression following treatment with a demethylating reagent.

## Discussion

In the present study, genome-wide DNA methylation and RNA expression profiling was conducted to identify novel genes associated with cervical cancer. An integrated analysis of the methylation and expression profiles was conducted and differentially-expressed genes regulated by DNA methylation were selected. To determine whether these genes were associated with cervical cancer, CCDB and GENT were used. The selected genes were also confirmed as novel epigenetic markers by treating HeLa cells with a demethylating reagent and measuring DNA methylation status and RNA expression.

To identify novel genes associated with cervical cancer, the majority of studies have focused on genome-wide RNA expression analysis (23,24). DNA methylation is another natural mechanism of regulating specific gene expression (7). Using high-throughput analysis, including microarray-based systems, global DNA methylation analyses have been used to investigate the DNA methylation status in the whole genome (13). However, not all DNA methylation regulates RNA expression associated with cancer initiation and progression. DNA methylation and RNA expression should be analyzed on a genome-wide scale simultaneously using the same clinical samples. Thus, an integrated analysis of DNA methylation and RNA expression profiles was conducted in the present study using a genome-wide microarray-based system. As shown in Fig. 1, the selected genes showed a negative correlation for methylation and expression profiles. This result supports the fact that the selected genes showing differential expression between normal and tumor tissues are regulated by the DNA methylation status. Two publicly available databases were used to analyze the candidate genes selected from the present study. Firstly, to identify the novelty of selected genes with cervical cancer, a search was applied to observe whether the candidate genes were already reported in the CCDB or not. A total of 15 of the selected 19 genes were identified as novel genes associated with cervical cancer (Table I). Secondly, to overcome the shortage of sample numbers, the expression of the selected genes in cervical cancer was further analyzed using the microarray database (GENT). A total of 16 of the selected 19 genes showed positive expression patterns with the present data (Table I). These results from the two databases indicate that the selected genes may be novel genes associated with cervical cancer and involved in the progression of cervical cancer.

Gene ontology analysis showed that the first cluster covered two categories, the cell junction and the plasma membrane (Table II). Four genes, *CAMK2N1*, *CGNL1*, *DLC1* and *SYT11*, the expression of which was lower in the tumor tissues than in the normal tissues, were enriched in the cell junction. Generally, proteins associated with the cell junction suppress cell proliferation and stimulate cell differentiation. Downregulation of these genes has been linked to epithelial-mesenchymal transition and cancer (25). Seven genes, including three additional genes, *FAT1*, *MUC4* and *TPM1*, were categorized in the plasma membrane. It has been reported that the *MUC* family genes, *MUC1*, *MUC16* and *MUC4*, are upregulated in cervical squamous cell carcinoma (26,27). Similarly to this previous study, the *MUC4* gene was shown to be upregulated by hypomethylation in the pre-cancer tissues compared with that in the normal tissues in the present study (Table I). Based on these analyses, the selected genes were clustered into enrichment categories associated with cancer progression.

Treatment with demethylating reagent decreased the methylation of selected genes and increased the demethylation (Fig. 2). Furthermore, gene expression was increased. These results support the fact that the selected genes are regulated by DNA methylation. Methylation of *ALDH1A3* was originally reported in lung cancer and was identified as a methylation marker for other types of cancer, including breast, prostate, colon and brain tumors (28,29). However, to the best of our knowledge, there have been no studies regarding the methylation of *ALDH1A3* in cervical cancer. Two other genes, *PPP1R3C* and *CAMK2N1*, were downregulated by hypermethylation in cervical cancer. The epigenetic regulation of these genes was validated in the present study (Fig. 2). Based on the findings of a previous study, the *PPP1R3C* gene is downregulated in cervical cancer (30); its methylation status has been reported in melanoma, colon and prostate cancers, but not in cervical cancer (31,32). Similar to the present results, the *PPP1R3C* gene is highly downregulated in cervical cancer, as per the GENT database. These results are consistent with the present data, including the downregulation and hypermethylation of the *PPP1R3C* gene. The *CAMK2N1* gene is a calcium/calmodulin-dependent protein kinase II inhibitor 1, and its expression is negatively correlated with the progression of human colon cancer. The silencing of *CAMK2N1* expression increases tumor growth and cell cycle progression (33). There have been no studies examining *CAMK2N1* and methylation in cancer. It can be postulated that the *CAMK2N1* gene is regulated by DNA methylation, since CpG islands are located in the first exon and intron in the *CAMK2N1* gene, according to the University of California Santa Cruz genome browser (http://genome.ucsc.edu). Based on these data, *CAMK2N1* may play important roles in cervical cancer progression through epigenetic regulation.

In conclusion, in the present study, 19 genes associated with cervical cancer were identified showing differential RNA expression by DNA methylation using the integrated analysis of data from RNA expression and DNA methylation arrays. The present study also determined that a number of the 19 genes have not previously been reported in association with cervical cancer and validated the epigenetic regulation of three genes, *ALDH1A3*, *PPP1R3C* and *CAMK2N1*, by MS-PCR and RT-quantitative PCR. Taken together, these results suggest that the selected genes may be involved in the progression or initiation of cervical cancer through the differential expression by DNA methylation. The functional role of these genes or their potential as novel DNA methylation markers remains to be examined.

## Figures and Tables

**Table I:** Differentially-expressed and methylated genes in tumor tissues compared with the normal tissues of patients with cervical cancer.

Gene	Accession number	Name	Δ value	Fold change	CCDB	GENT
Downregulated genes by hypermethylation in cancer						
*SYT11*	NM_152280.2	Synaptotagmin XI	0.32	−2.70	NA	Down
*CAMK2N1*	NM_018584.4	Calcium/calmodulin-dependent protein kinase II inhibitor 1	0.28	−4.95	NA	Up
*ALDH1A3*	NM_000693.1	Aldehyde dehydrogenase 1 family, member A3	0.27	−3.08	NA	Not changed
*PRRX1*	NM_006902.3	Paired related homeobox 1, transcript variant pmx-1a	0.25	−3.18	NA	Down
*PPP1R3C*	NM_005398.3	Protein phosphatase 1, regulatory (inhibitor) subunit 3C	0.22	−6.18	Down	Down, highly
*MAP1LC3A*	NM_181509.1	Microtubule-associated protein 1 light chain 3α, transcript variant 2	0.22	−3.14	NA	Down
*PHF21B*	NM_138415.1	PHD finger protein 21B	0.20	−2.06	NA	Down, highly
*TPM1*	NM_001018004.1	Tropomyosin 1 (α), transcript variant 7	0.20	−2.07	NA	Down, highly
*PPP1R14A*	NM_033256.1	Protein phosphatase 1, regulatory (inhibitor) subunit 14A	0.20	−2.91	NA	Down, highly
Upregulated genes by hypomethylation in cancer						
*PLOD2*	NM_000935.2	Procollagen-lysine, 2-oxoglutarate 5-dioxygenase 2, transcript variant 2	−0.25	2.66	Up	Up, highly
*FAT1*	NM_005245.3	FAT tumor suppressor homolog 1 (*Drosophila*)	−0.23	3.44	NA	NA
*EXT1*	NM_000127.2	Exostoses (multiple) 1	−0.22	2.40	NA	Up
*TMEM44*	NM_138399.3	Transmembrane protein 44, transcript variant 1	−0.21	3.02	NA	Up
Downregulated genes by hypermethylation in pre-cancer						
*SNCAIP*	NM_005460.2	Synuclein, alpha interacting protein	0.23	−2.39	NA	Down
*A2M*	NM_000014.4	α-2-macroglobulin	0.21	−2.22	Up	Down
*CGNL1*	NM_032866.3	Cingulin-like 1	0.21	−3.14	NA	Down, highly
*DLC1*	NM_024767.2	Deleted in liver cancer 1, transcript variant 3	0.20	−2.45	Down	Down, highly
*RNF150*	NM_020724.1	Ring finger protein 150	0.20	−2.18	NA	Down, highly
Upregulated genes by hypomethylation in pre-cancer						
*MUC4*	NM_018406.3	Mucin 4, cell surface-associated, transcript variant 1	−0.21	2.94	NA	Up

CCDB, cervical cancer database; GENT, gene expression database across normal and tumor tissues; NA, not applicable.

**Table II:** Functional annotation clustering (gene ontology) of 19 selected genes showing five clusters.

Annotation cluster	Enrichment category	Count	P-value	False discovery rate^a^
1				
GOTERM_CC_FAT	Cell junction	4	1.70×10^−2^	8.20×10^−1^
GOTERM_CC_FAT	Plasma membrane part	7	2.20×10^−2^	6.80×10^−1^
GOTERM_CC_FAT	Plasma membrane	7	2.10×10^−1^	9.30×10^−1^
2				
GOTERM_CC_FAT	Cytoskeletal part	4	8.10×10^−2^	8.10×10^−1^
GOTERM_CC_FAT	Cytoskeleton	4	1.90×10^−1^	9.20×10^−1^
GOTERM_CC_FAT	Intracellular non-membrane-bound organelle	4	5.60×10^−1^	1.00
GOTERM_CC_FAT	Non-membrane-bound organelle	4	5.60×10^−1^	1.00
3				
SP_PIR_KEYWORDS	Membrane	9	2.00×10^−1^	9.60×10^−1^
GOTERM_CC_FAT	Integral to plasma membrane	3	3.80×10^−1^	9.90×10^−1^
GOTERM_CC_FAT	Intrinsic to plasma membrane	3	3.90×10^−1^	9.90×10^−1^
UP_SEQ_FEATURE	Topological domain: Cytoplasmic	5	4.00×10^−1^	1.00
UP_SEQ_FEATURE	Transmembrane region	6	5.10×10^−1^	1.00
SP_PIR_KEYWORDS	Transmembrane	6	5.20×10^−1^	9.90×10^−1^
GOTERM_CC_FAT	Intrinsic to membrane	7	6.00×10^−1^	1.00
UP_SEQ_FEATURE	Topological domain: Extracellular	3	7.50×10^−1^	1.00
GOTERM_CC_FAT	Integral to membrane	6	7.60×10^−1^	1.00
4				
UP_SEQ_FEATURE	Glycosylation site: N-linked (GlcNAc)	6	3.50×10^−1^	1.00
SP_PIR_KEYWORDS	Signal	5	3.60×10^−1^	9.90×10^−1^
UP_SEQ_FEATURE	Signal peptide	5	3.70×10^−1^	1.00
SP_PIR_KEYWORDS	Glycoprotein	6	3.80×10^−1^	9.90×10^−1^
UP_SEQ_FEATURE	Disulfide bond	3	7.70×10^−1^	1.00
SP_PIR_KEYWORDS	Disulfide bond	3	7.80×10^−1^	1.00
5				
SP_PIR_KEYWORDS	Metal-binding	4	5.40×10^−1^	9.90×10^−1^
GOTERM_MF_FAT	Metal ion binding	5	8.00×10^−1^	1.00
GOTERM_MF_FAT	Cation binding	5	8.10×10^−1^	1.00
GOTERM_MF_FAT	Ion binding	5	8.20×10^−1^	1.00
GOTERM_MF_FAT	Transition metal ion binding	3	8.90×10^−1^	1.00

a Using the Benjamini method. κ similarity (similarity term overlap, 3; similarity threshold, 0.50); classification (initial group membership, 3; final group membership, 3; multiple linkage threshold, 0.50); enrichment thresholds (ease 1.0). Enrichment scores for the five annotation clusters were as follows: 1, 1.37; 2, 0.58; 3, 0.33; 4, 0.33; and 5, 0.12.

**Table III:** Primers sequences for methylation-specific polymerase chain reaction.

Gene	Nature of sequence primer	Sense	Antisense	Product size, bp
*CAMK2N1*	Methylated	AATTAGGAGGGGACGTTAAAATC	ACATATATCCCTAACAAACAACGAA	185
	Unmethylated	GAATTAGGAGGGGATGTTAAAATT	ACATATATCCCTAACAAACAACAAA	186
*ALDH1A3*	Methylated	GTTCGTTTATTGACGAAATTTTTTC	AAAAACCGTACGCTTCTACGAC	134
	Unmethylated	TTGTTTATTGATGAAATTTTTTTGG	ACAAAAAACCATACACTTCTACAAC	135
*PPP1R3C*	Methylated	ATTTGTTTTTAAGTACGTGATTCGA	GATACCCAAATAACTCTCTACACGTC	153
	Unmethylated	ATTTGTTTTTAAGTATGTGATTTGA	AATACCCAAATAACTCTCTACACATC	153
